# Comment on Detection of Mycotoxins in Patients with Chronic Fatigue Syndrome. *Toxins* 2013, *5*, 605–617

**DOI:** 10.3390/toxins8110322

**Published:** 2016-11-07

**Authors:** John W. Osterman

**Affiliations:** Montreal, Canada; johnwosterman@hotmail.com; Tel.: 1-514-941-4919

The paper by Brewer et al. entitled “Detection of Mycotoxins in Patients with Chronic Fatigue Syndrome. *Toxins* 2013, *5*, 605–617” is so methodologically flawed that it should never have been published in the scientific literature [1]. 

In this paper, the authors measure the presence of mycotoxins in the urine of 112 patients suffering from chronic fatigue syndrome (CFS). These finding are then compared to urine samples from 55 healthy control subjects “... with no history of exposure to WDB (water damaged buildings) or moldy environment...” (sic). Not surprisingly, there were more people from the CFS group with mold exposure than in the comparison group. These results are not surprising because, BY DEFINITION, the control group had no history of exposure to mold. By purposely choosing a control group with no history of mold exposure, the authors have statistically rigged their results in such a way that only a positive relationship will be found when compared to the CFS group. 

Using the same approach, the authors could test urine from their CFS patients for the presence of caffeine metabolites and compare the results to urine from a group not exposed to caffeinated beverages; they would find more caffeine metabolites in the CFS group for the same methodological reasons, the control group having been purposely selected to be not exposed. The same would be true for nicotine metabolites in the CFS patients’ urine using urine from non-smokers as a comparison group or comparing urinary animal protein metabolites from the CFS group to animal protein metabolites in urine from vegetarians. The results from these studies would show a positive but erroneous association between CFS and caffeine, nicotine and animal protein. The same is true for the relationship that Brewer et al. purportedly found in this study of CFS and mold. The findings from this study are misleading and meaningless. 

This study is an example of extreme selection bias and is akin to showing that men are shorter than women by comparing the height of an average group of men to that of women on the national basketball team! 

Given the mountain of “junk” science on the Internet, I feel that a credible on-line scientific journal must ensure rigorous methodological standards for the papers it publishes. Such was not the case for this paper.
